# Biological Fitness Cost, Demographic Growth Characteristics, and Resistance Mechanism in Alpha-Cypermethrin-Resistant *Musca domestica* (Diptera: Muscidae)

**DOI:** 10.3390/biology12071021

**Published:** 2023-07-19

**Authors:** Abdulwahab M. Hafez, Naeem Abbas

**Affiliations:** Pesticides and Environmental Toxicology Laboratory, Department of Plant Protection, College of Food and Agriculture Sciences, King Saud University, Riyadh 11451, Saudi Arabia

**Keywords:** house fly, pyrethroid resistance, detoxification enzymes, age-stage life table, demographic traits

## Abstract

**Simple Summary:**

The house fly is a pest of animals and humans that has developed resistance to alpha-cypermethrin, a commonly used pyrethroid insecticide used to control medically important pests in Saudi Arabia. This study demonstrated that the alpha-cypermethrin selected strain of house fly had a 405.93-fold increase in resistance to alpha-cypermethrin compared with the alpha-cypermethrin–susceptible strain; the increased resistance was attributed to the presence of glutathione S-transferases, specific esterases, and cytochrome P450 monooxygenases. The alpha-cypermethrin resistant strain exhibited lower relative fitness (0.50), longevity, survival rate, life expectancy, reproductive values, intrinsic rate of increase, net reproductive rate, fecundity, maternity, and finite rate of increase, along with shorter larval, female preadult, and adult durations than the susceptible strain. These results demonstrated that alpha-cypermethrin resistance may pose fitness costs in house flies. These findings will aid in the development of rational house fly control methods.

**Abstract:**

*Musca domestica* L., a pest of animals and humans, has developed resistance to alpha-cypermethrin, a pyrethroid insecticide commonly used to control medically important pests in many countries, including Saudi Arabia. We investigated the mechanism underlying the development of alpha-cypermethrin resistance and life history characteristics of alpha-cypermethrin–susceptible (Alpha-SS) and alpha-cypermethrin-resistant (Alpha-RS) *M. domestica* using the age-stage, two-sex life table theory, which is crucial for developing a future rational management strategy and minimizing the negative effects of alpha-cypermethrin on the environment. Our results showed that Alpha-RS *M. domestica* had a 405.93-fold increase in resistance to alpha-cypermethrin relative to Alpha-SS *M. domestica*. This increase in the resistance toward insecticide was attributed to metabolic enzymes, such as glutathione S-transferases, specific esterases, and cytochrome P450 monooxygenases. Furthermore, Alpha-RS *M. domestica* exhibited lower relative fitness (0.50), longevity, survival rate, life expectancy, reproductive values, intrinsic rate of increase, net reproductive rate, fecundity, maternity, and finite rate of increase, along with shorter larval, female preadult, and adult durations than Alpha-SS *M. domestica*, indicating fitness costs associated with most parameters. However, no significant differences were found between the strains in the following parameters: egg, pupa, and male preadult durations; adult preoviposition, total preoviposition, and oviposition periods; female ratio; and total generation time. Additionally, Alpha-RS *M. domestica* had a markedly lower intrinsic rate of increase, net reproductive rate, and finite rate of increase than Alpha-SS *M. domestica*. The results of this study suggest that alpha-cypermethrin resistance may lead to dominant fitness costs in *M. domestica*. Overall, these findings will aid in the development of rational control strategies for *M. domestica* as well as help to reduce pesticide pollution.

## 1. Introduction

*Musca domestica* L. (Diptera: Muscidae), the common house fly, is a globally distributed insect pest that transmits diseases to humans and animals [1,2]. The adult stage of *M. domestica* not only causes annoyance but also serves as a carrier for approximately 200 pathogens responsible for several life-threatening diseases, such as avian influenza, diarrhea, bovine respiratory diseases, typhoid fever, aspergillosis, tuberculosis, cholera, poliomyelitis, and hepatitis [2,3,4,5]. These pathogens are attached to the body of *M. domestica* adults either through direct contact or while feeding on garbage and animal waste in filthy habitats [5,6]. Subsequently, the pathogens are transmitted to humans and animals through physical contact when the flies enter sanitary settings [7].

Various measures, including the maintenance of proper waste hygiene around dairies and homes, chemical control, and biological agents such as predators and parasitoids, are taken to manage *M. domestica* [1]. Among these measures, chemical control is a common practice due to its knockdown effects. Alpha-cypermethrin, a synthetic pyrethroid insecticide, is used worldwide to control various insect pests, including *M. domestica* [8,9,10]. Unfortunately, overreliance on alpha-cypermethrin can lead to resistance development, increased control costs, and environmental pollution [11,12]. In fact, alpha-cypermethrin resistance has previously been observed in *M. domestica*, *Bactrocera oleae* Rossi., *Rhipicephalus microplus* Canestrini, and *Stomoxys calcitrans* L. [11,13,14,15]. As a result, increasing the dose of insecticide has become inevitable, which exerts adverse effects on the environment.

Insecticide resistance mechanisms are often associated with metabolic detoxification, reduced cuticular penetration, and target site insensitivity [16,17]. Metabolic detoxification and/or sodium channel-facilitated target site insensitivity have been recognized as major mechanisms underlying the resistance to insecticides in *M. domestica* [18,19,20,21,22]. Pyrethroid resistance in *M. domestica* is often linked with mutations (*kdr*) as well as increased activities of cytochrome P450 monooxygenases, glutathione S-transferases, and/or general esterases [17,22,23,24,25].

Fitness is an individual’s ability to reproduce in a particular environment and pass on their reproductive traits to subsequent generations [26,27]. Fitness costs are exhibited in insecticide-resistant individuals of pest strains that are less fit than insecticide-susceptible individuals in the absence of insecticides. These costs tremendously contribute to delaying the evolution of insecticide resistance and reducing its extent in the absence of insecticides [28]. The discontinuation of insecticide selective pressure can prevent the occurrence and spread of insecticide resistance when insecticide-resistant pests exhibit dominant fitness costs [29]. Insecticide resistance affects the fitness of insects, resulting in lower survival rates, fecundity (number of eggs per female), finite or intrinsic rates of increase, and net reproductive rates, along with longer developmental durations and generation times in insecticide-resistant pests than in insecticide-susceptible pests [29,30]. The fitness costs of resistance to various insecticide classes have been described in *M. domestica* [29,31], *Dysdercus koenigii* Fabricius [32], *Aedes aegypti* L. [33], *Bemisia tabaci* (Gennadius) [34], and *Oxycarenus hyalinipennis* Costa [35]. These insecticide-resistant strains have shown unfavorable biological and demographic growth characteristics compared with their susceptible counterpart strains. However, the fitness cost of alpha-cypermethrin resistance in *M. domestica* is still unknown.

Understanding fitness costs and mechanisms of insecticide resistance is crucial for the development of effective strategies to manage insecticide resistance. In our previous study, we assessed the resistance risk, quantitative genetics, and cross-resistance patterns in alpha-cypermethrin-resistant *M. domestica* [14]. However, the fitness costs and the mechanisms of alpha-cypermethrin resistance in *M. domestica* remain unknown. Therefore, in this study, the fitness costs of alpha-cypermethrin-resistant (Alpha-RS) and alpha-cypermethrin-susceptible (Alpha-SS) *M. domestica* were investigated based on the age-stage, two-sex life table theory. Furthermore, the mechanisms of alpha-cypermethrin resistance were explored using three synergists.

## 2. Materials and Methods

### 2.1. Strains of M. domestica

The rearing protocol for *M. domestica* strains is outlined in our previous studies [10,14,36]. Briefly, we collected a population of *M. domestica* from a dairy facility in Dirab, Riyadh, Saudi Arabia (24.49° N, 46.60° E) and divided it into two groups: Alpha-SS, which was maintained for 41 generations with no insecticide exposure; and Alpha-RS, which was continuously exposed to various concentrations of alpha-cypermethrin (90–1000 ppm) for 41 generations. Selection pressure was maintained by exposing subsequent generations to increased concentrations of alpha-cypermethrin while ensuring sufficient survival of adults for offspring production. The median lethal concentration (LC_50_) of alpha-cypermethrin in the field strain (90 ppm) was used for the first eight generations. In each generation, approximately 1000 mixed-sex adults (2–3 days old) were screened with alpha-cypermethrin using a feeding method (IRAC method# 026) [37], and mortality data were recorded after exposure to insecticide for 48 h. Surviving adults were transferred to rearing cages (40 × 40 cm^2^) for obtaining the next generation. Both strains were reared in the laboratory under controlled conditions: 27 °C ± 2 °C, 65% ± 5% relative humidity, and a 12:12 h light:dark photoperiod.

### 2.2. Chemicals

Alpha-cypermethrin (Alphaquest, 100EC, 10% a.i., Astrachem Company, Dammam, Saudi Arabia) was used for bioassay and selection experiments. Synergistic bioassays were performed using common inhibitors of major metabolic enzymes: piperonyl butoxide (PBO; 97%, Rhawn Reagent Co., Ltd., Shanghai, China), which is a cytochrome P450 monooxygenases inhibitor; S,S,S-tri-n-butyl phosphorotrithioate (DEF; Chem. Service Inc., West Chester, PA, USA), an esterase-specific inhibitor; and diethyl maleate (DEM; 97%, Tokyo Chemical Industry Co., Ltd., Tokyo, Japan), a glutathione S-transferase inhibitor.

### 2.3. Concentration–Response Bioassay

Concentration–response bioassays were conducted to determine the resistance levels in the tested *M. domestica* following the selection pressure by alpha-cypermethrin over 41 generations. We used a concentration–response bioassay that has been described in our previous studies [10,14,36], with slight modifications. Briefly, the toxicity of alpha-cypermethrin against *M. domestica* adults was assessed using a feeding bioassay. Seven alpha-cypermethrin concentrations, whose mortality rates ranged from >0% to <100%, were prepared in 20% sugar solution through serial dilution and replicated three times. Each desired concentration was prepared by diluting a specific amount from the stock solution of alpha-cypermethrin into the relevant volume of the 20% sugar solution. We used alpha-cypermethrin concentrations ranging from 0.977 to 62.5 μg mL^−1^ and from 125 to 8000 μg mL^−1^ for Alpha-SS and Alpha-RS, respectively. For each replicate, concentration, and bioassay, 10, 30, and 210 mixed-sex adults were used, respectively. Thirty adults were used as controls, with ten adults included in each replicate. These adults were starved for 2 h in perforated plastic jars (11 cm diameter; 15 cm height) prior to the bioassay. Cotton wicks (3 cm in length) soaked in each insecticide concentration were placed in Petri dishes (9 cm in diameter); these Petri dishes were kept in plastic jars for feeding the insects. For the control group, cotton wicks soaked in 20% sugar solution were provided for feeding. Concentration–response bioassays were performed under the laboratory conditions mentioned in Section 2.1. Mortality was assessed after 48 h of exposure, considering the fast action of alpha-cypermethrin.

### 2.4. Synergism Experiment

Synergism bioassays were conducted to identify the possible mechanism of alpha-cypermethrin resistance in the tested *M. domestica* following the selection pressure by alpha-cypermethrin over 41 generations. In these synergism bioassays, analytical-grade acetone (Fisher Scientific, Loughborough, UK) was used to dissolve PBO, DEF, and DEM. The resulting solutions were mixed with serially diluted concentrations of alpha-cypermethrin. The concentrations of alpha-cypermethrin that were mixed with PBO, DEF or DEM ranged from 0.977 to 62.5 μg mL^−1^ and from 62.5 to 4000 μg mL^−1^ for Alpha-SS and Alpha-RS, respectively. Based on the results of preliminary tests, a nonlethal concentration of 2 mg/L for PBO and DEF was used against both Alpha-RS and Alpha-SS *M. domestica*, and a nonlethal concentration of 0.5 mg/L was used for DEM against both strains. The control consisted of acetone mixed with deionized water only. Synergism bioassays were performed under the laboratory conditions mentioned in Section 2.1. Mortality was recorded after 48 h of exposure, and the synergism ratios (SRs) were calculated as the LC_50_ of alpha-cypermethrin alone divided by the LC_50_ of alpha-cypermethrin plus PBO, DEF, or DEM. The 95% confidence limits (CLs) were determined using the method described by Robertson et al. [38].

### 2.5. Assessment of Life Tables of Alpha-RS and Alpha-SS M. domestica

The life table parameters of Alpha-SS and Alpha-RS *M. domestica* were studied to assess the fitness cost of alpha-cypermethrin resistance. Beginning with the 41st generation, three replications of 150 randomly selected freshly hatched first instar larvae (50 larvae per replicate) from Alpha-SS and Alpha-RS *M. domestica* were used as experimental populations. The larvae were placed in 1000 mL glass beakers containing artificial diet (described in detail by Abbas and Hafez [14] and protected with a fine mesh cloth to prevent larvae from escaping. Larvae were allowed to pupate in glass jars, and the durations of larval and pupal stages as well as mortality rates were recorded for both strains. Within 24 h, the emerged males and females were paired in plastic jars (15 cm height; 11 cm diameter). Adult diet (1 mg of dry powdered milk and 1 mg of sugar) and water-soaked cotton wicks (~3 cm) were placed in separate Petri dishes and provided to adult flies for feeding. To examine fecundity, 50 ♂ × 50 ♀ (1:1 ratio) and 30 ♂ × 30 ♀ (1:1 ratio) pairs of Alpha-SS and Alpha-RS, respectively, were made based on the availability of adults. One pair of *M. domestica* was used as one replication for both strains. After 2 days of adult pairing, Petri dishes containing larval diet were provided for egg laying. Petri dishes were removed from jars daily, and eggs were counted using a fine hair brush. All life history parameters were recorded for both strains under the aforementioned laboratory conditions.

The demographic life table parameters were calculated using methods outlined in our previous studies [1,32,39].

“The age-stage survival rate” (*s_xj_*), i.e., the probability that a newly laid egg will survive to age *x* and stage *j*, was calculated using the method described by Chi and Liu [40]. The age-specific survival rate (*l_x_*), i.e., the pooled probability that a newly laid egg will survive to age *x*, was determined as follows:

(1) $$l_{x}=\sum_{j=1}^{m}s_{xj}.$$

Stage differentiation is not possible because *l_x_* represents the pooled survival rates of all stages at age *x*. The age-specific fecundity (*m_x_*), i.e., the mean fecundity of all individuals at age *x*, was determined as follows:

(2) $$m_{x}=\frac{\sum_{j=1}^{m}s_{xj}f_{xj}}{\sum_{j=1}^{m}s_{xj}},$$

where *f_xj_* is the mean fecundity of individuals at age *x* and stage *j*, and *m* is the number of stages. The net reproductive rate (*R*_0_), i.e., the total number of offspring that an individual can produce during its lifetime, was determined as follows:

(3) $$R_{0}=\sum_{x=0}^{\infty }l_{x}m_{x}.$$

The intrinsic rate of increase (*r*), i.e., the population growth rate when the time approaches infinity and the population reaches the stable age-stage distribution, was calculated using the Euler–Lotka equation [41,42] with an age index of zero [43] as follows:

(4) $$\sum_{x=0}^{\infty }{e^{-r(x+1)}l}_{x}m_{x}=1.$$

The finite rate of increase (λ) was determined using the following equation:

λ = *e^r^*. (5)

The mean generation time (*T*), i.e., the length of time that a population requires to increase *R*_0_-fold in its size at the stable age-stage distribution, was calculated as follows:

(6) $$T=\frac{ln\;R_{0}}{r}$$

The life expectancy (*e_xj_*), i.e., the expected duration of time that an individual of age *x* and stage *j* will survive after age *x*, was determined using the following equation:

(7) $$e_{xj}=\sum_{\;i=x}^{n}\sum_{y=j}^{m}s_{iy}^{’},$$

where $s_{iy}^{’}$ is the probability that an individual of age *x* and stage *j* will survive until age *i* and stage *y* [44]. The reproductive value (*v_xj_*), i.e., the value of an individual at age *x* and stage *j* to future offspring [45], was calculated as follows:

(8) $$v_{xj}=\frac{e^{r(x+1)}}{s_{xj}}\sum_{i=x}^{\infty }e^{-r(i+1)}\sum_{y=i}^{m}s_{iy}^{’}f_{iy}$$

The curves for *l_x_*, *s_xj_*, *m_x_*, age-stage specific maternity (*l_x_m_x_*), *e_xj_*, and *v_xj_* were plotted using Sigma Plot 11.0. 

Relative fitness (*Rf*) was calculated as follows:

(9) $$Rf=\frac{R_{0}ofAlpha\text{-}RS}{R_{0}ofAlpha\text{-}SS}.$$

### 2.6. Data Analysis

Mortality and life table data analyses were conducted using methods outlined in our previous studies [1,14,32,39]. Probit analysis was performed using Polo Plus Software to analyze mortality data [46] and obtain LC_50_ values, fiducial limits (FLs), slopes (±standard errors (SEs)), and chi-square values (χ^2^). Abbott’s formula [47] was used to correct for control treatment mortality. Resistance ratios (RRs) were calculated by dividing the LC_50_ of alpha-cypermethrin in Alpha-RS *M. domestica* by that in Alpha-SS *M. domestica*. SRs were calculated by dividing the LC_50s_ of alpha-cypermethrin alone in Alpha-SS and Alpha-RS *M. domestica* by the LC_50s_ of alpha-cypermethrin +PBO, DEF, or DEM in Alpha-SS and Alpha-RS *M. domestica*. The 95% CLs of RRs and SRs were assessed using the method described by Robertson et al. [38], and RRs were considered significantly different if the CLs were not 1. The life table data were analyzed using the TWO-SEX-MS Chart program using 100,000 replicates and a paired bootstrap test [48] to calculate the means and SEs. The differences in life table traits between Alpha-RS and Alpha-SS *M. domestica* were compared using confidence intervals (CIs) calculated via the paired bootstrap test [44,49,50]. A significant difference among life history traits of strains was indicated by CIs that did not include the value 0 at *p* ≤ 0.05.

## 3. Results

### 3.1. Toxicity and Resistance of Alpha-RS M. domestica to Alpha-Cypermethrin

After 41 generations of selection in the laboratory, Alpha-RS had 405.93-fold increase in resistance to alpha-cypermethrin relative to Alpha-SS (Table 1).

### 3.2. Effect of Synergists on Alpha-Cypermethrin Toxicity in Alpha-RS and Alpha-SS M. domestica

The synergists PBO, DEF, and DEM did not exhibit any synergistic effect on alpha-cypermethrin toxicity in Alpha-SS *M. domestica* (Table 2). However, PBO, DEF, and DEM significantly reduced alpha-cypermethrin LC_50_ values in Alpha-RS *M. domestica* from 1530.36 ppm to 412.18 ppm, 254.23 ppm, and 439.71 ppm, respectively, with SRs of 3.71, 6.02, and 3.48, respectively. These findings suggested that mechanisms of alpha-cypermethrin resistance in Alpha-RS *M. domestica* may be associated with specific esterases, glutathione S-transferases, and cytochrome P450 monooxygenases (Table 2).

### 3.3. Biological and Demographic Parameters of Alpha-RS and Alpha-SS M. domestica

The egg duration, pupal duration, ♂ preadult duration, adult preoviposition period, total preoviposition period, oviposition period, generation time, and female ratio were not significantly different between Alpha-RS and Alpha-SS *M. domestica*. However, the larval duration, ♀ preadult duration, overall adult duration, ♂ total longevity, ♀ total longevity, and overall total longevity were significantly shorter in Alpha-RS *M. domestica* than in Alpha-SS *M. domestica*. Additionally, reproductive female ratio, fecundity, *r*, and λ were considerably lower in Alpha-RS *M. domestica* than in Alpha-SS *M. domestica*. *Ro* of Alpha-RS *M. domestica* was negatively affected when compared with that of Alpha-SS *M. domestica*. Compared with Alpha-SS *M. domestica*, the relative fitness value of Alpha-RS *M. domestica* was 0.50 (Table 3).

### 3.4. Age-Stage Survival and Reproduction Parameters of Alpha-RS and Alpha-SS M. domestica

**Figure 1:**
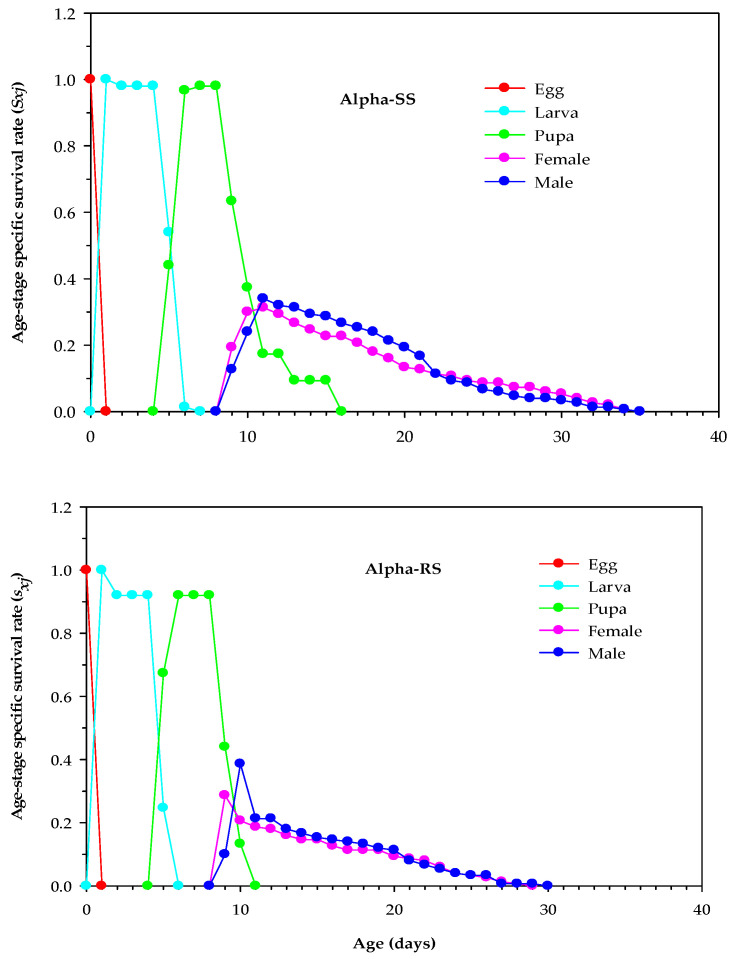
Age-stage survival rates (*s_xj_*) in alpha-cypermethrin-susceptible (Alpha-SS) and alpha-cypermethrin-resistant (Alpha-RS) strains of *Musca domestica.*

Compared with Alpha-SS *M. domestica*, the *s_xj_* of male, female, pupa, and larva were negatively affected in Alpha-RS *M. domestica* (*p* < 0.05). However, the peak value of *s_xj_* for egg in Alpha-RS *M. domestica* was similar to that in Alpha-SS *M. domestica* (*p* > 0.05). The highest *s_xj_* values in the larva (0.92), pupa (0.92), female (0.21), and male (0.21) of Alpha-RS *M. domestica* were lower than those in the larva (1.00), pupa (0.98), female (0.31), and male (0.34) of Alpha-SS *M. domestica* (Figure 1).

**Figure 2:**
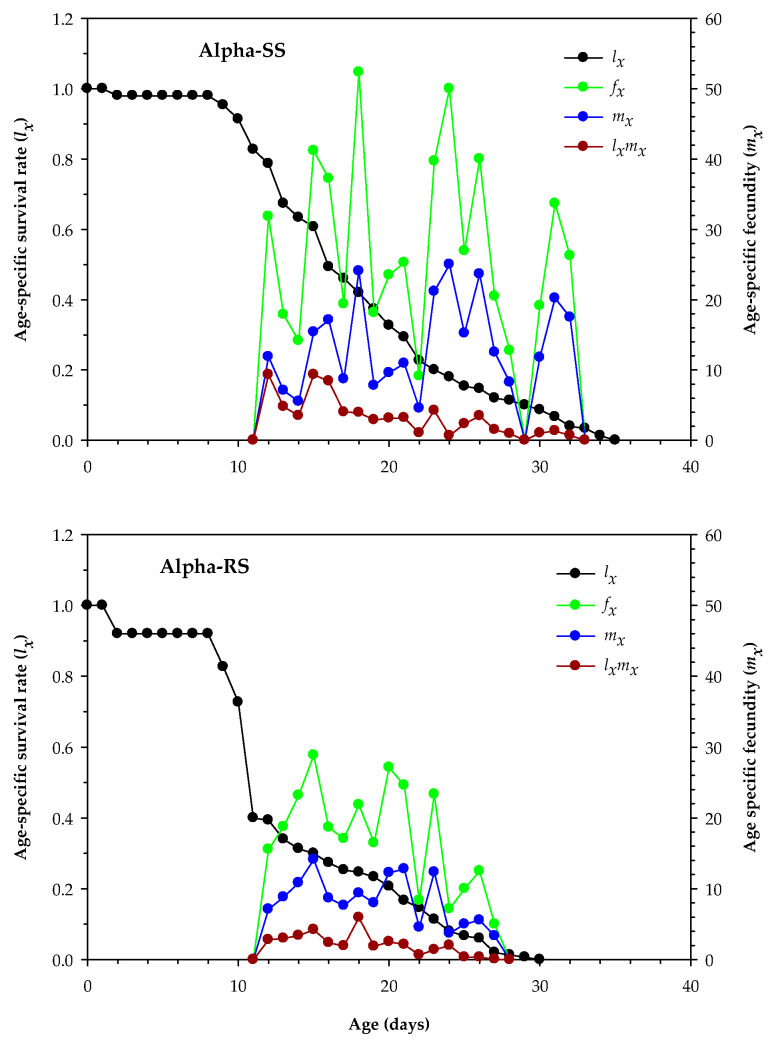
Age-specific survival rate (*l_x_*), age-specific female fecundity (*f_x_*), age-specific fecundity of the total population (*m_x_*), and age specific maternity (*l_x_m_x_*) in the alpha-cypermethrin-susceptible (Alpha-SS) and alpha-cypermethrin-resistant (Alpha-RS) *Musca domestica.*

The peak value of *l_x_* in Alpha-RS *M. domestica* was similar to that in Alpha-SS *M. domestica* (*p* > 0.05). However, the maximum peak of age-stage specific female fecundity (*f_x_*), *m_x_*, and *l_x_m_x_* peak values were significantly lower in Alpha-RS *M. domestica* than those in Alpha-SS *M. domestica* (*p* < 0.05). A comparison of the *l_x_* curves for Alpha-RS and Alpha-SS *M. domestica* revealed that Alpha-SS *M. domestica* aged 9–35 days and Alpha-RS *M. domestica* aged 9–30 days had low *l_x_* (Figure 2). Alpha-RS *M. domestica* aged 15 days had an *f_x_* of 28.86 eggs/female/day, which was lower than that in Alpha-SS *M. domestica* aged 18 days (53.25 eggs/female/day) (Figure 2). The maximum peak of *m_x_* (25 eggs/day) was observed in Alpha-SS *M. domestica* females aged 24 days, which was higher than the *m_x_* peak (14.11 eggs/day) in Alpha-RS *M. domestica* females aged 15 days (Figure 2). Both Alpha-SS *M. domestica* (9.33 offspring/day) and Alpha-RS *M. domestica* (4.23 offspring/day) exhibited maximum *l_x_m_x_* peaks (9.33 offspring/day and 4.23 offspring/day, respectively) when they were 15 days old (Figure 2).

**Figure 3:**
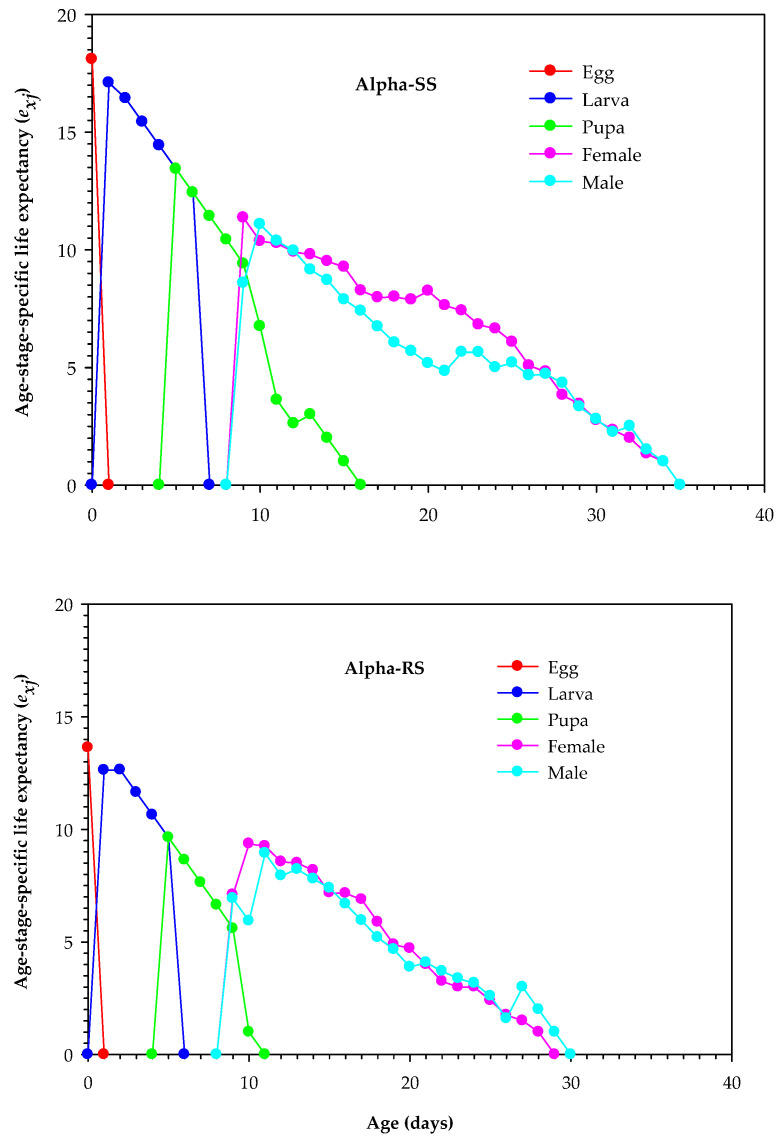
Age-stage life expectancy (*e_xj_*) in the alpha-cypermethrin-susceptible (Alpha-SS) and alpha-cypermethrin-resistant (Alpha-RS) strains of *Musca domestica.*

The *e_xj_* of egg, larva, and pupa of female and male Alpha-RS *M. domestica* was worse than that of Alpha-SS *M. domestica* (*p* < 0.05). Alpha-SS *M. domestica* had a higher maximum *e_xj_* value for egg (18.10 days) than Alpha-RS *M. domestica* (13.63 days). The peak *e_xj_* value for larva was 17.10 days in Alpha-SS *M. domestica*, which was higher than that of Alpha-RS *M. domestica* (12.64 days). Similarly, the peak *e_xj_* value for pupa of Alpha-SS *M. domestica* (13.43 days) was significantly higher than that of Alpha-RS *M. domestica* (9.64 days). In both species, male and female *M. domestica* had significantly different peak *e_xj_* values, with Alpha-SS *M. domestica* male and female having a longer life expectancy than Alpha-RS *M. domestica* (11.36 days and 11.08 days, respectively) (Figure 3).

**Figure 4:**
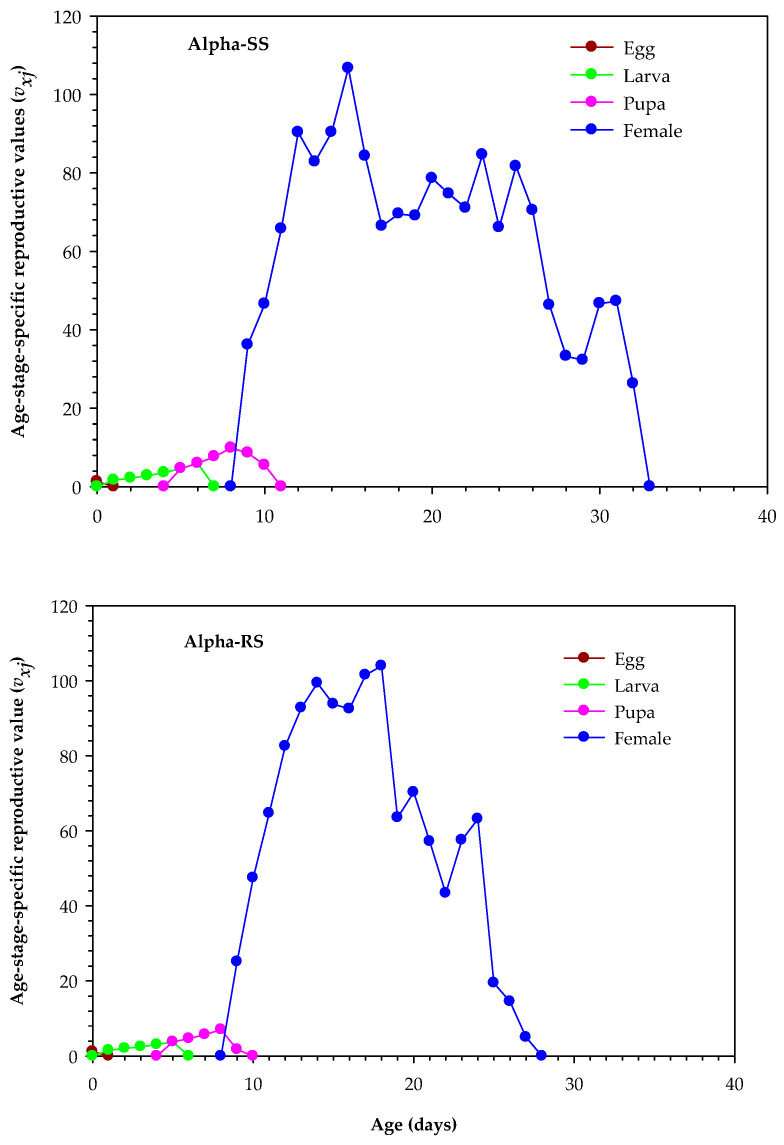
Age-stage reproductive values (*v_xj_*) in the alpha-cypermethrin-susceptible (Alpha-SS) and alpha-cypermethrin-resistant (Alpha-RS) *Musca domestica.*

Alpha-SS *M. domestica* had higher maximum *v_xj_* values than Alpha-RS *M. domestica* for egg, larva, pupa, and female, with values of 1.29, 5.96, 9.86, and 106.68 per day, respectively, compared with *v_xj_* values of 1.23, 3.78, 7.04, and 103.95 per day, respectively, for Alpha-RS *M. domestica* (*p* < 0.05). The durations were also shorter in Alpha-RS *M. domestica* than in Alpha-SS *M. domestica* (Figure 4).

## 4. Discussion

Alpha-cypermethrin is a relatively modern pyrethroid insecticide used in Saudi Arabia for controlling medically important pest species, including *M. domestica* [10,14]. In this study, Alpha-RS *M. domestica* had 405.93-fold resistance to alpha-cypermethrin after 41 generations of selection than Alpha-SS *M. domestica*. This rapid increase in the resistance of *M. domestica* to alpha-cypermethrin suggests that continuous exposure to this insecticide enhanced their insecticide resistance. Enzymatic detoxification is the most likely reason for the increase in alpha-cypermethrin resistance, as found in this study. The involvement of metabolically mediated resistance to insecticides can be preliminarily assessed using enzyme inhibitors such as DEF, PBO, and DEM [17,51]. The current findings revealed that the LC_50_ of alpha-cypermethrin in Alpha-RS *M. domestica* was significantly decreased in the presence of PBO, DEF, or DEM compared to when alpha-cypermethrin was used alone, indicating a possible involvement of the specific esterases, glutathione S-transferases, and cytochrome P450 monooxygenases in the development of alpha-cypermethrin resistance in Alpha-RS *M. domestica*. This result suggests that specific esterases, glutathione S-transferases, and cytochrome P450 monooxygenases can be used to mitigate the toxicity of alpha-cypermethrin. Previous studies have indicated that *M. domestica* has developed pyrethroid resistance through cytochrome P450–mediated detoxification [18,21,52,53]. Our results are in agreement with those of Abbas et al. [17] and Khan et al. [54], who found that cytochrome P450 monooxygenases and specific esterases were involved in mitigating the toxicity of lambda-cyhalothrin and deltamethrin in *M. domestica*. However, Zhang et al. [55] reported no involvement of cytochrome P450 monooxygenases and glutathione S-transferases in mitigating the toxicity of beta-cypermethrin in *M. domestica*.

It is crucial to understand the fitness costs that accompany insecticide resistance in pest strains for developing effective strategies to manage insecticide resistance [26,28]. Insecticide-resistant individuals often exhibit unfavorable biological traits [39]. In this study, Alpha-RS *M. domestica* had a relative fitness of 0.50 when compared with Alpha-SS *M. domestica*; it also had shorter larval and adult durations, female preadult period, lower male and female total longevity, smaller reproductive female ratio, and lower fecundity than Alpha-SS, which shows the fitness costs associated with alpha-cypermethrin resistance. Previous studies also suggest that if the selection pressure of alpha-cypermethrin is discontinued for a period, the population of Alpha-RS *M. domestica* does not increase as rapidly as that of Alpha-SS *M. domestica*. Previous studies have also detected fitness costs in different insecticide-resistant strains, such as *M. domestica* [29,31,56,57,58], *Phlebotomus papatasi* (Scopoli) [59], *Ae. aegypti* [60,61], *O. hyalinipennis* [62,63], *Plutella xylostella* L. [64], *Chrysodeixis includens* Walker [65], *Rhopalosiphum padi* L. [66], and *Ceratitis capitata* (Wiedemann) [67].

Demographic growth features, such as *r*, *λ*, and *Ro*, are indicators of potential population growth in certain environments. Changes in these features can disrupt the growth rate of insects [39]. In this study, *r*, *λ*, and *Ro* were significantly lower among Alpha-RS *M. domestica* than among Alpha-SS *M. domestica*, indicating the effect of alpha-cypermethrin selection on the reproduction and growth of *M. domestica*. The decrease in the number of eggs produced by a female in Alpha-RS *M. domestica* may be responsible for the reduced *r*, *λ*, and *Ro* values and subsequent decrease in relative fitness. Previous studies have also reported significantly reduced *r* and *Ro* in pyrethroid-resistant *M. domestica*, *C. includens*, *C. capitata*, *Anopheles funestus* Giles, *Ae. aegypti*, *P. papatasi*, and *O. hyalinipennis* compared with their pyrethroid-susceptible counterparts [29,59,61,62,65,67,68]. Additionally, *s_xj_*, *m_x_*, *l_x_m_x_*, *e_xj_*, and *v_xj_* were markedly reduced in Alpha-RS *M. domestica* compared with Alpha-SS *M. domestica*, indicating fitness costs associated with alpha-cypermethrin resistance. Consistent with our results, previous studies have reported significant reductions in these age-stage, two-sex life table traits in different insecticide-resistant insect pests [32,69]. Due to time constraints, we were not able to perform the required crosses of Alpha-SS and Alpha-RS to ascertain whether the fitness costs were due to a heterosis effect relative to deleterious resistant alleles or genetic drift in the heterozygotes leading to recovery of biological parameters [30,70]. In general, our results support the common notion that insecticide-resistant strains exhibit fitness costs compared with their insecticide-susceptible counterparts. Furthermore, our study provides a comprehensive understanding of fitness traits in both Alpha-RS and Alpha-SS *M. domestica*.

The presence of deleterious life table parameters in Alpha-RS *M. domestica* highlights the negative impact of resistant alleles on the fitness and mechanisms of insect resistance, which may result from a trade-off between resources and energy. The selection pressure of insecticides, including alpha-cypermethrin, on pest strains requires significant consumption of resources and energy [30] contributing to fitness costs and adverse biological and reproduction parameters in Alpha-RS *M. domestica*. To address this issue, temporary withdrawal of alpha-cypermethrin, and the use of insect growth regulators, which are effective against *M. domestica* [1], can help in restoring the susceptibility of these insects to pyrethroids and aid successful *M. domestica* management. Rational insect resistance management plans, including limited use of insecticides, biological control agents, and cultural practices, should also be considered while planning *M. domestica* management.

## 5. Conclusions

After 41 generations, Alpha-RS *M. domestica* developed a 405.93-fold higher resistance to alpha-cypermethrin than Alpha-SS *M. domestica*, primarily via specific esterases, cytochrome P450 monooxygenases, and glutathione S-transferases. However, fitness costs were observed in this strain, as evinced by its unfavorable life history parameters compared with those of Alpha-SS *M. domestica*, indicating that the development of alpha-cypermethrin resistance could be delayed by withdrawing the use of insecticide for a given period of time. Hence, implementing resistance management strategies, such as alternating the use of alpha-cypermethrin with new insecticides exhibiting different chemistry, could delay alpha-cypermethrin resistance development. Moreover, integrated pest management (such as chemical, cultural, and biological control) should be implemented for the management of *M. domestica*. These findings provide valuable insights for developing effective pyrethroid resistance management against *M. domestica*, ultimately preserving the environmental fauna by minimizing the use of higher insecticide doses.

## Figures and Tables

**Table 1:** Toxicity and resistance of *Musca domestica* against alpha-cypermethrin.

Strain	LC_50_ (ppm) ^a^	95% Fiducial Limits	Fit of Probit Line				RR (95% CL) ^b^
			Slope ± SE	*χ* ^2^	Df	*p*	
Alpha-SS (G_41_)	3.77	2.52–5.28	1.33 ± 0.18	0.66	5	0.98	1
Alpha-RS (G_41_)	1530.36	1193.24–1992.02	1.98 ± 0.23	3.42	5	0.64	405.93 (260.73–631.56)

^a^ Median lethal concentration. ^b^ Resistance ratio (RR) and 95% confidence limits (CLs).

**Table 2:** Effects of synergists on the toxicity of alpha-cypermethrin in Alpha-SS and Alpha-RS *Musca domestica.*

Strain	Insecticide	LC_50_ (ppm) ^a^	95% Fiducial Limits (ppm)	Fit of Probit Line				SR (95% CL) ^b^
				Slope ± SE	*χ* ^2^	df	*p*	
Alpha-SS (G_41_)	Alpha-cypermethrin	3.77	2.52–5.28	1.33 ± 0.18	0.66	5	0.98	1
	+PBO	3.57	2.40–4.96	1.36 ± 0.19	2.47	5	0.78	1.06 (0.64–1.76)
	+DEF	3.09	2.16–4.16	1.58 ± 0.21	2.73	5	0.74	1.22 (0.75–1.99)
	+DEM	3.28	2.10–4.66	1.27 ± 0.18	2.68	5	0.75	1.15 (0.68–1.95)
Alpha-RS (G_41_)	Alpha-cypermethrin	1530.36	1193.24–1992.02	1.98 ± 0.23	3.42	5	0.64	1
	+PBO	412.18	258.79–559.98	2.37 ± 0.43	1.04	5	0.95	3.71 (2.39–5.77) *
	+DEF	254.23	168.98–354.18	1.61 ± 0.22	1.32	5	0.93	6.02 (3.87–9.37) *
	+DEM	439.71	328.48–585.10	1.63 ± 0.20	1.19	5	0.94	3.48 (2.37–5.11) *

^a^ Median lethal concentration. ^b^ Synergism ratio (SR) and 95% confidence limits (CLs). * SR differed significantly if the CLs did not include 1.

**Table 3:** Fitness cost and demographic life table features of alpha-cypermethrin-resistant (Alpha-RS) and counterpart susceptible (Alpha-SS) *Musca domestica* strains.

Parameters	Alpha-SS (Mean ± SE)	Alpha-RS (Mean ± SE)	95% CI	*p*
Egg duration (d)	1.00 ± 0.00 a	1.00 ± 0.00 a	-	>0.05
Larval duration (d)	4.56 ± 0.04 a	4.27 ± 0.04 b	0.18–0.41	<0.0001
Pupal duration (d)	4.24 ± 0.04 a	4.28 ± 0.04 a	−0.08–0.16	0.52
Adult duration (d)	10.47 ± 0.65 a	6.65 ± 0.60 b	2.07–5.55	<0.0001
♂ Preadult duration (d)	9.95 ± 0.10 a	9.74 ± 0.06 a	−0.02–0.44	0.08
♀ Preadult duration (d)	9.55 ± 0.10 a	9.07 ± 0.04 b	0.28–0.69	<0.0001
♂ Total longevity (d)	20.02 ± 0.85 a	15.93 ± 0.76 b	1.86–6.31	<0.0001
♀ Total longevity (d)	20.47 ± 1.02 a	16.30 ± 0.94 b	1.44–6.88	0.003
Overall total longevity (d)	20.23 ± 0.65 a	16.10 ± 0.59 b	2.40–5.86	0.00
APOP (d)	3.54 ± 0.17 a	3.76 ± 0.17 a	−0.24–0.69	0.35
TPOP (d)	13.08 ± 0.23 a	12.76 ± 0.17 a	−0.24–0.88	0.27
Oviposition period (d)	4.64 ± 0.47 a	3.76 ± 0.38 a	−0.31–2.07	0.15
Female ratio (%)	0.47 ± 0.05 a	0.44 ± 0.05 a	−0.11–0.16	0.71
Reproductive female ratio (%)	0.76 ± 0.06 a	0.54 ± 0.07 b	0.03–0.41	0.02
Fecundity (eggs produced per female)	205.18 ± 32.10 a	114.13 ± 22.11 b	14.28–167.25	0.02
*r* (d^−1^)	0.25 ± 0.01 a	0.21 ± 0.01 b	1.08–7.92	0.01
*λ* (d^−1^)	1.29 ± 0.01 a	1.23 ± 0.02 b	1.38–9.92	0.01
T (d)	16.84 ± 0.35 a	17.13 ± 0.27 a	−0.58–1.17	0.51
*Ro* (offspring individual^−1^)	69.76 ± 13.48 a	35.00 ± 7.99 b	3.98–65.40	0.03
*Rf*	1.00	0.50	/	/

♂, males; ♀, females; APOP, adult preoviposition period; TPOP, total preoviposition period; *r*, intrinsic rate of increase; *λ*, finite rate of increase; T, generation time; *Ro*, net reproductive rate; *Rf*, relative fitness; d, days; CI, confidence interval; SE, standard error (calculated using bootstrapping with resampling 100,000 times). Differences between strains were determined using the paired bootstrap test (*p* < 0.05). Means with different lowercase letters in rows are significantly different.

## Data Availability

The data presented in this study are available from the corresponding author on a reasonable request.
